# Duloxetine, a Balanced Serotonin-Norepinephrine Reuptake Inhibitor, Improves Painful Chemotherapy-Induced Peripheral Neuropathy by Inhibiting Activation of p38 MAPK and NF-κB

**DOI:** 10.3389/fphar.2019.00365

**Published:** 2019-04-09

**Authors:** Jing Meng, Qiuyan Zhang, Chao Yang, Lu Xiao, Zhenzhen Xue, Jing Zhu

**Affiliations:** ^1^Jiangsu Key Laboratory for Pharmacology and Safety Evaluation of Chinese Materia Medica, Department of Pharmacy, Nanjing University of Chinese Medicine, Nanjing, China; ^2^Jiangsu Province Key Laboratory of Anesthesia and Analgesia Application Technology, Xuzhou Medical University, Xuzhou, China; ^3^Departments of Neurology and Neuroscience, Johns Hopkins University School of Medicine, Baltimore, MD, United States

**Keywords:** chemotherapy-induced peripheral neewopathy (CIPN), duloxetine, oxaliplatin (OXA), paclitaxel (PTX), eripheral neuropathic pain, dorsal root ganglia (DRG)

## Abstract

Chemotherapy-induced peripheral neuropathy (CIPN) is a severe, toxic side effect that frequently occurs in anticancer treatment and may result in discontinuation of treatment as well as a serious reduction in life quality. The CIPN incidence rate is as high as 85–90%. Unfortunately, there is currently no standard evidence-based CIPN treatment. In several clinical trials, it has been reported that duloxetine can improve CIPN pain induced by oxaliplatin (OXA) and paclitaxel (PTX); thus, The American Society of Clinical Oncology (ASCO) recommends duloxetine as the only potential treatment for CIPN. However, this guidance lacks the support of sufficient evidence. Our study shows that duloxetine markedly reduces neuropathic pain evoked by OXA or PTX. Duloxetine acts by inhibiting the activation of p38 phosphorylation, thus preventing the activation and nuclear translocation of the NF-κB transcription factor, reducing the inflammatory response and inhibiting nerve injury by regulating nerve growth factor (NGF). Furthermore, in this study, it is shown that duloxetine does not affect the antitumor activity of OXA or PTX. This study not only provides biological evidence to support the use of duloxetine as the first standard CIPN drug but will also lead to potential new targets for CIPN drug development.

## Introduction

A major dose-limiting complication of chemotherapy is chemotherapy-induced peripheral neuropathy (CIPN). The greatest contributors to CIPN are taxanes (e.g., paclitaxel) and platinum-based (e.g., oxaliplatin) treatments (Krukowski et al., 2015). Paclitaxel (PTX) can effectively treat several of the most common cancers including breast cancer, lung cancer, and ovarian cancer (Ewertz et al., 2015; Cetinkaya-Fisgin et al., 2016; Hopkins et al., 2016). Oxaliplatin (OXA), a third-generation diaminocyclohexane (DACH) platinum agent, is used as a first-line chemotherapy in combination with 5-fluorouracil to treat resectable and advanced colorectal cancer (Ta et al., 2006). However, these chemotherapy drugs can induce painful neuropathy at an incidence rate as high as 85–90%. Patients with CIPN experience sensory dysfunction including allodynia, hyperalgesia, dysesthesia, and paranaesthesia (Makker et al., 2017). These symptoms can lead to chronic disabilities that persist despite dose reduction and discontinuation of treatment. Recent studies have evaluated chemotherapy-related neuropathic pain. However, the mechanisms by which chemotherapy drugs cause neuropathy are not well understood, which severely limits development of novel therapeutic methods and drugs (Taillibert et al., 2016).

Duloxetine is a balanced serotonin-norepinephrine reuptake inhibitor. Recent clinical trials have shown that it can effectively control painful CIPN induced by OXA and PTX (Piccolo and Kolesar, 2013; Okuma et al., 2016). Thus, the American Society of Clinical Oncology recommends duloxetine as the only treatment for CIPN. However, the scientific evidence for this guidance is limited and the mechanism of action of duloxetine has not been characterized (Hershman et al., 2014). In our study, we evaluated the neuroprotective effects of duloxetine using CIPN models induced by paclitaxel or oxaliplatin *in vivo* and *in vitro*. For *in vitro* experiments, methyl-thiazolyl-tetrazolium (MTT) and ViaLight Plus kit were used to determine cell viability, and immunofluorescence staining was used to measure axon length. *In vivo* experiments demonstrated that CIPN was partially improved by duloxetine in mice that developed pain hypersensitivity and exhibited changes in cytokine levels and intra-epidermal nerve fiber (IENF) density.

Furthermore, we investigated the potential mechanism of action of duloxetine-mediated modulation of neuropathic pain in dorsal root ganglia (DRG) of OXA- or PTX-treated mice. Previous studies have shown that the inflammatory response plays an important role in modulation of neuropathic pain (Austin and Moalem-Taylor, 2010; Vallejo et al., 2010; Zhang et al., 2013). Other studies have suggested that the inflammatory response is also an important factor in CIPN reduction (Boyette-Davis and Dougherty, 2011; Boyette-Davis et al., 2011). Recent studies showed that MAPKs and NF-κB signaling participate in development of OXA- and PTX-induced neuropathy (Li et al., 2015; Chen et al., 2016; Yeo et al., 2016). In addition, a previous study showed increased extracellular signal related kinase (ERK1/2) and p38 signaling in the DRG. However, c-Jun N terminal kinase and PI3K-Akt signaling were not increased (Li et al., 2015). Our study showed that duloxetine improved peripheral nerve fiber density and hypersensitivity behaviors, and decreased levels of OXA- or PTX-induced NF-κB and p-p38 proteins.

## Materials and Methods

### Duloxetine Efficacy – *In vitro* Experiments

#### Drugs

Oxaliplatin (Sigma, United States) was dissolved in sterile water to a final concentration of 3 mM. PTX (100 mM, Sigma, United States) and duloxetine (10 mM, Sigma, China) were dissolved in dimethyl sulfoxide (DMSO). All drugs were then diluted with culture medium to the indicated working standard concentrations.

#### Cell Culture and Treatments

Dorsal root ganglia were dissected from Sprague-Dawley rats within 1 day of birth. DRG were incubated with 3 mg/mL of collagenase type I solution (Worthington Biochemical Corporation) at 37°C for 50 min. The suspension was centrifuged at 1,500 rpm for 3 min and then suspended in Neurobasal medium (Gibco, United States) supplemented with 10% fetal bovine serum (FBS, Gibco, United States), 1% penicillin/streptomycin (Gibco, United States), 0.5 mM L-glutamine (Gibco, United States), 0.2% glucose, 2% B27 supplement (Gibco) and 10 ng/mL of glial cell line-derived neurotrophic factor (Peprotech, United States). All sterile tissue culture plates were previously coated with Laminin (10 μg/mL, Invitrogen, United States) and Poly-L-Lysine (150 μg/mL, Sigma-Aldrich, United States).

Dorsal root ganglia neurons were cultured in 96-well plates or in 24-well plates at a density of 5,000 cells per well and incubated in a humidified 37°C, 5% CO_2_ incubator. Cells were incubated for 24 h for all experiments. The cells were exposed to PTX (300 nM) for 24 h or OXA (3 μM) for 48 h with different concentrations of duloxetine in culture medium containing 2% serum. For untreated cells, only complete medium was added.

#### Axon Length Measurement

Dorsal root ganglia neurons were seeded on poly-L-lysine coated glass and grown in 24-well plates. After drug treatment, neurons were fixed with 4% paraformaldehyde solution and stained with anti-βIII-tubulin antibody (1:2,000, Abcam, United States) at 4°C overnight. Neurons were visualized using a fluorescence microscope (IX71, Olympus, Japan). Random sampling was used to determine axon length.

#### Assay of DRG Neuronal Cell Injury

Based on terminal deoxynucleotidyl transferase-mediated dUTP nick end labeling (TUNEL), the Cell Death Detection Kit (KGA7062, KeyGen, China) was used to measure nuclear damage. In addition, cultured DRG cells were seeded on poly-L-lysine-coated glass and grown in 24-well plates. After experimental manipulations, neurons were fixed with 4% paraformaldehyde for 30 min and permeabilised with 0.1% Triton X-100. Then, the cells were incubated with TUNEL reaction mixture in the dark for 60 min at 37°C according to the manufacturer’s instructions. Neurons were visualized by fluorescence microscopy (IX71, Olympus, Japan).

#### Apoptosis Assay

Apoptosis was evaluated using the Alexa V-FITC/propidium iodide (PI) Apoptosis Detection Kit (KGA107, KeyGen, China). Cells were grown in 6-well plates and digested with trypsin. After washing twice with PBS, cells were resuspended in 500 μl of binding buffer and stained with Annexin V-FITC/PI for 15 min on ice. The proportion of apoptotic cells was determined by flow cytometry (Accuri C6, BD, United States). Apoptotic cells were indicated by positive Annexin V staining.

#### Effects of Duloxetine on the Antitumor Activity of OXA or PTX

The cancer cell lines SUM-159 (breast cancer) and HT-29 (colorectal cancer) were plated in 96-well plates to assess the effects of duloxetine on OXA- and PTX-induced cell death using the MTT assay. SUM-159 cells were incubated in high glucose DMEM (Hyclone, United States) and HT-29 cells were incubated in RMPI-1640 medium (Gibco, United States) each containing 1% penicillin/streptomycin (Gibco, United States) and 10% FBS (Gibco, United States). Varying concentrations of duloxetine, 300 nM PTX, or 30 μM OXA were added to the wells. For the MTT assay, we added 10 μL of MTT (0.5 mg/mL) to each well. After 4 h the supernatant was removed and the formazan crystals were dissolved in 110 μL of DMSO. Absorbance was measured at 490 nm using a Multimode Plate Reader (Tecan, Switzerland).

#### ViaLight Plus

For these experiments, DRG cells were also grown in 96-well white view plates. ATP levels were evaluated by the ViaLight Plus Cell Proliferation and Cytotoxicity Bioassay Kit (Lonza, LT07-121). After 24 h or 48 h incubation with the different regents, cells were washed twice with PBS (100 μL per well). To this, 50 μL of the cell lysis reagent was added, and cells were incubated in the orbital shaker for 5 min at 800 rpm. After this time, 100 μL of AMR Plus was added, and the plate was incubated for 2 min in the dark before measurement with the Multimode Plate Reader (SPARK10M, Tecan, Mannedorf, Switzerland).

### Duloxetine Efficacy – *In vivo* Studies

#### Experimental Animals

All procedures involving animals were performed in accordance with the ethical guidelines established by the International Association for the Study of Pain (Zimmermann, 1983), and the protocols were approved by the Animal Committee of Nanjing University of Chinese Medicine (Approval number, ACU171001). Male ICR mice weighing 18–22 g were used for these experiments (Nanjing, QingLongShan, China). All animals were kept in a humidity- and temperature-controlled environment and were maintained on a 12:12 h light–dark cycle and supplied with food and water *ad libitum*. All behavioral experiments were performed by an individual blinded to the treatment groups between 10 a.m–5 p.m.

#### CIPN Model and Drug Administration

Mice were randomly divided into six groups (*n* = 8 per group). A 6 mg/mL stock solution of PTX was prepared in cremophor:ethanol (1:1, v/v), then further diluted with 0.9% sterile saline to a final injection concentration of 2 mg/mL. OXA was dissolved in 5% glucose to a final concentration of 1 mg/mL. PTX (20 mg/kg) was injected intravenously (i.v.) into the tail vein on days 1, 3, and 5 (Zhu et al., 2013) and OXA (4 mg/kg) was injected intraperitoneally (i.p.) twice per week for a total of eight injections. Normal control (CONTROL) mice were injected with sterile saline, consistent with the drug-treated groups. There were three duloxetine treatment groups, including OXA with duloxetine (OXA+D), PTX with duloxetine (PTX+D), and duloxetine alone (D). Duloxetine was prepared in 0.9% sterile saline and administered at a dose of 30 mg/kg/day (i.p., 1 h prior to treatment with PTX or OXA). Each compound was administered at 0.1 mL/10 g of body weight. All precautions were taken to minimize animal suffering.

#### Behavioral Assessment

Behavioral tests were performed weekly (days 0, 7, 14, 21, and 28) after 5 h of drug administration. Prior to testing, the mice were allowed 30 min to acclimate to the testing apparatus. The experimenters were blinded to the drug treatment conditions during behavioral testing.

##### Mechanical withdrawal threshold test

Mechanical hyperalgesia was assessed using a Dynamic Plantar Aesthesiometer (DPA, Ugo Basile, Italy). Both the left and right hind paws were tested. The DPA automatically detected and recorded the latency time and the force of the withdrawal reflex during each paw withdrawal. The paw withdrawal latency test was performed in three times and the average value was calculated (Btesh et al., 2013; Russe et al., 2013). A movable force actuator was positioned below the plantar surface of the animal. The maximum force was set at 10 g to minimize pain in the animals and the ramp speed was 1 g/s. A Von Frey–type 0.5 mm filament exerted increasing force until the animal twitched its paw.

##### Thermal withdrawal latency test

Thermal hyperalgesia was tested on a plantar test (37370, Ugo Basile Plantar Test Apparatus, Italy) according to standard methods. Briefly, the plantar surface of the hind paw was exposed to a radiant heat source under a glass floor. Three measurements were taken during each test session. A 20-s cut-off time was set to avoid possible tissue damage. At 5-min intervals between consecutive tests, the hind paws were alternately tested (Wallace et al., 2007; Btesh et al., 2013). The average of three latency measurements was recorded as the result for each test.

##### Cold threshold test

Measurement of cold pain threshold was performed by a tail immersion test in ice water according to standard methods. Each mouse was lightly immobilized in a plastic fixator with their tail dipped in 4°C water and the tail withdrawal latency measurement was taken. To prevent pain in the tail, a 15-s cut-off time was set. The cold pain threshold test was repeated three times over 5-min intervals. The mean latency was used as the result (Chen et al., 2016; Kim et al., 2016).

#### Immunohistochemical Analysis and Quantification of IENF Density

Following experiments, all mice were sacrificed. Their plantar skin was dissected, fixed in PLP (Periodate-Lysine-Paraformaldehyde) solution overnight, and stored in 4°C. Prior to analysis, the samples were dehydrated with 30% sucrose overnight and embedded in OCT (Optimal Cutting Temperature compound) medium just prior to sectioning. Sections (30-μm-thick) were prepared from the footpad skin perpendicular to the epidermis using a sliding microtome. To ensure adequate and systematic sampling, every sixth section of each tissue was collected and a total of three frozen sections were used for immunohistochemistry. Sections were treated with 0.025 M potassium permanganate and 5% oxalic acid. Sections were blocked with 5% normal goat serum in non-fat dry milk and 10% Triton X-100 for 4 h, followed by incubation with primary antibody (the polyclonal neuron-specific marker anti-PGP9.5 at 1:200, ZSGB, China) overnight. After rinsing in TBS, sections were placed in biotinylated goat anti-rabbit IgG (1:1,000, Bioss, China) for 1 h, then in methanol/hydrogen peroxide/PBS for 30 min. Sections were incubated with the ABC Kit (Vector, PK-6100) for 1 h and then the SG Substrate Kit (Vector, PK-4700) until the desired darkness was reached. All slides were dehydrated once with ethanol and were treated with xylene following standard laboratory protocols. Images were captured at 400× magnification using a microscopy digital camera system (Olympus). Nerve fibers (PGP9.5 immunoreactive tissues) crossing the dermo-epidermal junction were quantified relative to the examined tissue length. At least four sections per group were measured and averaged (Ko et al., 2014; Areti et al., 2017).

### Western Blot

On day 28, at the end of the behavioral test session, a total of 48 animals were sacrificed for western blot assays. DRG tissues were homogenized in radio immunoprecipitation assay buffer. Total protein samples (30 μg) were separated by 10% SDS polyacrylamide gel electrophoresis at 100 V, then transferred to PVDF membranes. After the membranes were blocked for 1 h at room temperature in tris-buffered saline Tween-20 (TBST) containing 5% skim milk, they were incubated with primary antibodies specific for p38 MAPK (1:2,000, Cell Signaling Technology, Danvers, MA, United States), p-p38 MAPK (1:2,000, Cell Signaling Technology), ERK (1:2,000, Abcam, MA, United States), p-ERK (1:2,000, Cell Signaling Technology), NF-κB (1:1,000, Abcam), and GAPDH (1:4,000, Cell Signaling Technology) at 4°C overnight, followed by three washes with TBST solution, and incubation with secondary antibodies (1:4,000, Cell Signaling Technology) for 1h at room temperature (RT). Immunoreactivity was detected by chemiluminescence.

### Enzyme-Linked Immunosorbent Assay (ELISA)

Enzyme-linked immunosorbent assay was used to determine whether duloxetine regulated the cytokine levels [nerve growth factor (NGF), IL-6, TNF-α, and IL-1β] *in vivo*. Orbital blood samples were collected centrifuged (3500 rpm, 15 min), and the supernatant was stored at -80°C. Samples were assayed using mouse NGF (MaiBo, MBE10062), IL-6 (MaiBo, MBE10288), TNF-α (MaiBo, MBE10037), and IL-1β (MaiBo, MBE10289) ELISA kits according the manufacturer’s protocols. Optical density (OD) was recorded at 450 nm with correction at 570 nm using a Multimode Plate Reader (Tecan, Switzerland) (Kim et al., 2016; Makker et al., 2017).

### Immunofluorescence

Dorsal root ganglia neurons were seeded on poly-L-lysine coated glass and grown in 24-well plates. After drug treatment, neurons were fixed with 4% paraformaldehyde solution and stained with anti-NF-κB antibody (1:2,000, Abcam, United States) at 4°C overnight, followed by incubation with FITC-conjugated goat anti-mouse IgG (1:200, Jackson, United States) for 1 h at RT in the dark. Nuclei were stained for 5 min with diamidino-phenyl-indole (DAPI, Vector Laboratories). Neurons were visualized using a fluorescence microscope (IX71, Olympus, Japan).

### Analysis of Changes in Gene Expression Using Polymerase Chain Reaction (PCR)

The mRNA expression levels of IL-1β (Sangon Biotech, 5710324795, 5710324796), IL-6 (Sangon Biotech, 5710324791, 5710324792), and TNF-α (Sangon Biotech, 5710324793, 5710324794) were measured using a Real-Time PCR System (Applied Biosystems, Foster City, CA, United States). Briefly, total RNA was extracted from spinal cord DRG tissues using TRIZOL reagent (Invitrogen, United States) according to standard protocols, and cDNA was synthesized using ReverTra Ace qPCR RT Master Mix with gDNA Remover (Toyobo Co., Ltd., Life Science Department, Japan). Real-time PCR experiments were conducted following the protocol for TransStart Top Green qPCR SuperMix (TransGen Biotech). The IL-1β forward primer was 5′-TTC AGG CAG GCA GTA TCA CTC ATT G-3′ and the reverse primer was 5′-ACA CCA GCA GGT TAT CAT CAT CAT CC-3′. The IL-6 forward primer was 5′-AGA CTT CCA TCC AGT TGC CTT CTT G-3′ and the reverse primer was 5′-CAT GTG TAA TTA AGC CTC CGA CTT GTG-3′. The TNF-α forward primer sequence was 5′-GCG ACG TGG AAC TGG CAG AAG-3′ and the reverse primer was 5′-GAA TGA GAA GAG GCT GAG ACA TAG GC-3′. The GAPDH forward primer was 5′-TTC CTA CCC CCA ATG TAT CCG-3′ and the reverse primer was 5′-CAT GAG GTC CAC CAC CCT GTT-3′. The reaction conditions included denaturation at 94°C for 5 s followed by annealing and elongation at 60°C for 34 s. To achieve specificity and maximum efficiency, the binding positions of all primers were chosen to produce amplicons of 90–120 bp, and gel electrophoresis was performed to confirm the correct size of the primers and the absence of non-specific bands.

### Data Analysis

All data are expressed as the mean ± SEM and were analyzed by two-way ANOVA with Dunnett’s multiple comparison test using GraphPad Prism 5 software. Statistical significance was set at *P* < 0.05.

## Results

### Duloxetine Prevented Neurotoxicity and Axon Injury Induced by OXA or PTX in Primary DRG Neurons

We used primary rat DRG neuron-Schwann cells to examine the neurotoxicity of OXA and PTX. We also evaluated the neuroprotective capacity of duloxetine against OXA or PTX-induced neurotoxicity by measuring cell viability. Previous studies indicated that decreased ATP levels were related to axonal degeneration induced by PTX (Chen et al., 2007; Zhu et al., 2013; Cetinkaya-Fisgin et al., 2016), so we measured cellular ATP levels to evaluate duloxetine-induced neuroprotection. As seen in Supplementary Figure S1A, OXA caused approximately 55% toxicity at 3 μM. When DRG cells were treated with 3 μM OXA combined with varying concentrations of duloxetine (30 nM to 30 μM), duloxetine protected against OXA-induced neurotoxicity from 100 nM to 3 μM (Supplementary Figure S1B). As shown in Supplementary Figure S2A, we found that cell viability was decreased by 25% following exposure to 300 nM paclitaxel. Duloxetine protected against PTX-induced neurotoxicity at 300 nM (Supplementary Figure S2B).

**FIGURE 1:**
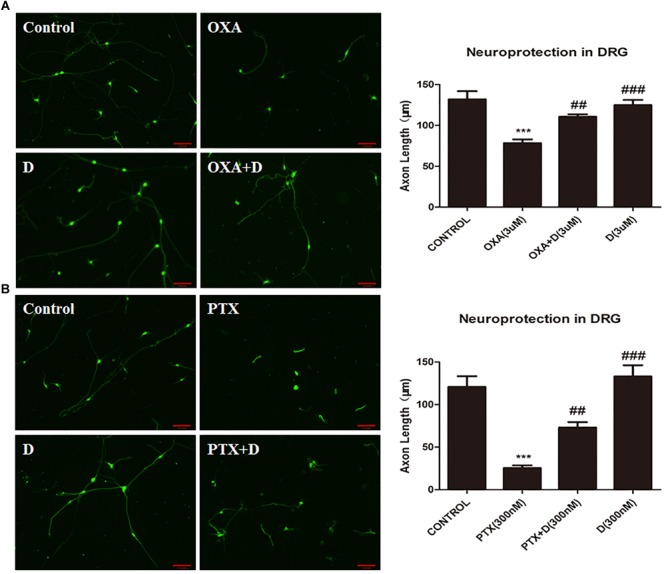
Effect of duloxetine on axonal injury caused by oxaliplatin or paclitaxel **(A)** Primary rat DRG neurons were grown and axons were extended for 24 h. Neurons were then treated with OXA (3 μM) or duloxetine (3 μM) for 48 h. Cells were then subjected to immunofluorescence staining, and axon length measurements were performed (^∗∗∗^*p* < 0.001 vs. control; ^##^*p* < 0.01; ^###^*p* < 0.001 vs. oxaliplatin alone). **(B)** Primary rat DRG neuronal cells were grown and axons were extended for 24 h. Neurons were then treated with PTX (300 nM) or duloxetine (300 nM) for 24 h. Cells were then subjected to immunofluorescence staining, and axon length measurements were performed (^∗∗∗^*p* < 0.001 vs. control; ^##^*p* < 0.01; ^###^*p* < 0.001 vs. paclitaxel alone). The results are expressed as the mean ± SEM (*n* = 10).

Furthermore, to show that duloxetine can prevent the axonal injury caused by OXA or PTX, we used immunofluorescence staining and measured axon lengths. Duloxetine provided partial protection against axonal degeneration induced by OXA or PTX (Figure 1A,B).

### Effect of Duloxetine on Neuronal Apoptosis in DRG Cells Treated by OXA or PTX

**FIGURE 2:**
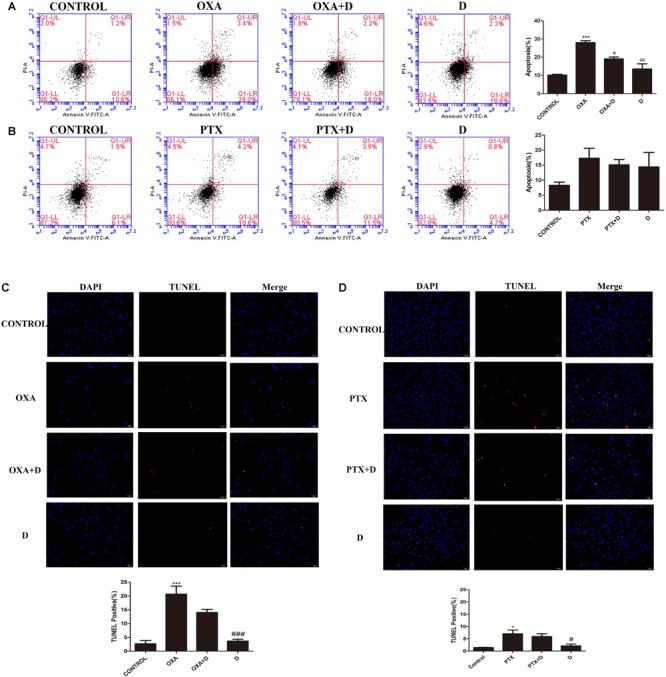
Effect of duloxetine on DRG neuronal apoptosis through flow cytometry and TUNEL assay. After growing in culture for 24 h, DRG neuronal cells were exposed to OXA (3 μM) with (or without) duloxetine (3 μM) for another 48 h **(A,C)** or exposed to PTX (300 nM) with (or without) duloxetine (300 nM) for another 24 h **(B,D)**. **(A,B)**:Cells were then double-stained with annexin V-FITC/PI. Annexin V-FITC fluorescence was measured with the FL1 channel, and PI fluorescence was measured with the FL3 channel. Representative pictures are from one of three independent experiments with similar results. **(C,D)** The cell death of DRG was examined by TUNEL assay. (^∗^*p* < 0.05, ^∗∗∗^*p* < 0.001 compared with control; ^#^*p* < 0.05, ^##^*p* < 0.01, ^###^*p* < 0.001 compared with model). The data are presented as mean ± SEM. Representative pictures are from one of three independent experiments with similar results (×200).

To determine whether duloxetine can play a neuroprotective role by reducing chemotherapy-induced apoptosis, terminal deoxynucleotidyl transferase dUTP nick end labeling (TUNEL) assay and flow cytometric analysis was performed. As shown in Figure 2, we found that OXA increased apoptosis in the DRG, and this increase was reversed by duloxetine. However, treatment with PTX had no significant effect on neuronal apoptosis compared to the control treatment. As such, our results indicated that OXA induced neuronal apoptosis and PTX damaged axons. Duloxetine partially blocked both effects. Administration of duloxetine alone did not significantly affect neuronal apoptosis compared to the control treatment. Analysis of annexin V/PI-stained cells by flow cytometry allowed for quantitation of cells that express (LR) annexin V-positive and PI-negative (early apoptotic), (UL) annexin V-negative and PI-positive (necrotic), (UR) annexin V-positive and PI-positive (late apoptotic), and (LL) annexin V-negative and PI-negative (live cells).

### Duloxetine Did Not Affect the Antitumor Activity of OXA or PTX

**FIGURE 3:**
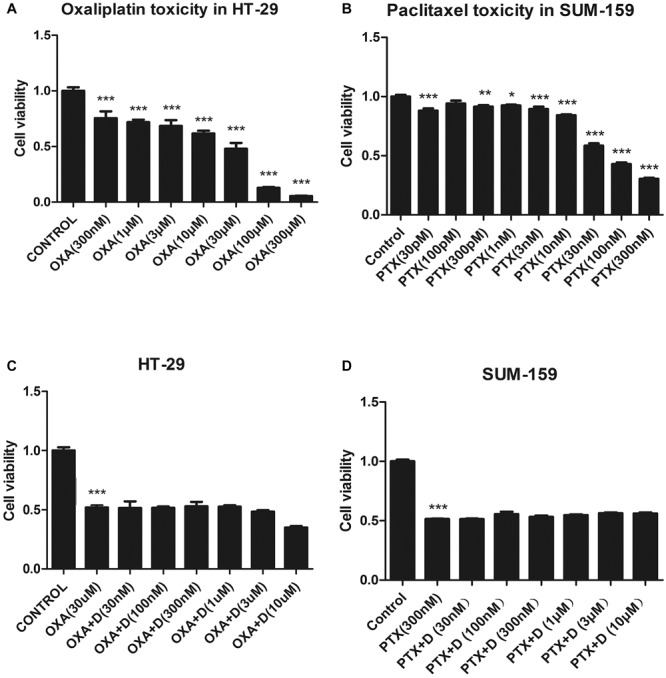
Effect of duloxetine on the anticancer activity of OXA or PTX *in vitro*. **(A,B)** When HT-29 or SUM-159 cancer cells were grown in medium, OXA or PTX reduced their cell viability by 20–80%. **(C,D)** Various concentrations of duloxetine together with OXA or PTX showed no significant changes in cell viability when compared to treatments with OXA or PTX alone. (^∗^*p* < 0.05, ^∗∗^*p* < 0.01,^∗∗∗^*p* < 0.001 vs. control). All the data are the mean ± SEM for each experiment (*n* = 4).

For duloxetine to be effective clinically, it should not affect the antitumor activity of OXA or PTX. We measured the viability of HT-29 (colon cancer cell line) and SUM-159 (breast cancer cell line) cells. Each of these cell lines was treated with various concentrations of OXA or PTX, and different concentrations of duloxetine, and cell viability was measured. As shown in Figure 3A, OXA caused >50% cell death in HT-29 cancer cells at or above 30 μM. Co-treatment with various concentrations of duloxetine with 30 μM OXA did not reduce the ability of OXA to kill HT-29 cancer cells (Figure 3C). As shown in Figure 3B, 300 nM PTX significantly reduced cancer cell viability by 50% compared to the control treatment. However, the antitumor effects of PTX were not altered by duloxetine (Figure 3D).

### Effects of Duloxetine on Pain Behavior in OXA- and PTX-Induced ICR Mice

**FIGURE 4:**
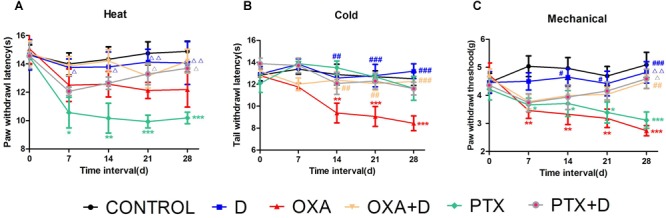
Partial prevention by duloxetine against OXA or PTX-induced peripheral neuropathy in ICR mice. **(A)** The effect of duloxetine on the heat withdrawal latency in mice treated with vehicle or chemotherapeutic drugs (OXA and PTX) was evaluated. **(B)** The effect of duloxetine on the cold threshold in mice treated with vehicle or chemotherapeutic drugs (OXA and PTX) was determined. **(C)** The effect of duloxetine on the mechanical withdrawal threshold in mice treated with vehicle or chemotherapeutic drugs (OXA and PTX) was assessed. (^∗^*p* < 0.05, ^∗∗^*p* < 0.01,^∗∗∗^*p* < 0.001 vs. control; ^#^*p* < 0.05,^##^*p* < 0.01, ^###^*p* < 0.001 vs. OXA; Δ*p* < 0.05, ΔΔ*p* < 0.01 vs. PTX). The data are presented as the mean ± SEM (*n* = 5–8).

To demonstrate duloxetine-induced protection against CIPN *in vivo*, we used OXA- and PTX-induced neuropathic pain models. The body weight of animals was presented in the Supplementary Figure S3. As shown in Figure 4A, PTX, but not OXA, caused significant heat hyperalgesia compared to the control group. Co-treatment with duloxetine prevented this effect. In contrast, OXA-treated mice, but not PTX-treated mice, exhibited cold hypersensitivity compared to the control group. Duloxetine treatment improved OXA-induced cold hypersensitivity (Figure 4B). Both OXA and PTX treatment led to mechanical hyperalgesia, and duloxetine increased the paw withdrawal threshold as demonstrated in experiments that used the DPA (Figure 4C). Duloxetine treatment alone did not induce any significant differences compared to control treatment.

### Duloxetine Partially Prevented Loss of IENF in OXA- or PTX-Induced Neuropathic Mice

**FIGURE 5:**
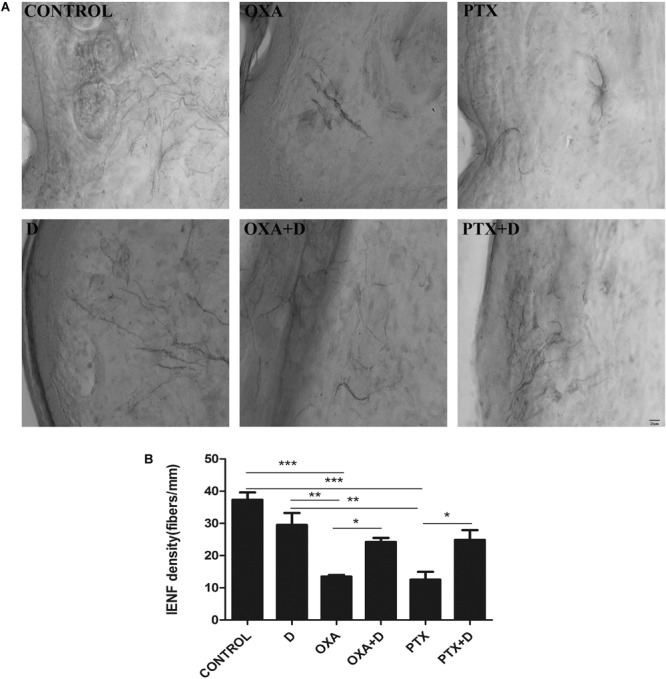
Effect of duloxetine on IENF retraction induced by OXA or PTX. Paw biopsies were obtained from the hind paws of mice when behavior tests were finished. Tissues were fixed and stained with antibodies (PGP9.5) for IENFs. **(A)** Representative images from six groups are shown. **(B)** IENF density = number of nerve fibers crossing the basement membrane/length of the basement membrane (mm). (Magnification × 400. ^∗^*p* < 0.05,^∗∗^*p* < 0.01,^∗∗∗^*p* < 0.001). The data are presented as the mean ± SEM (*n* = 5).

Both OXA and PTX induced significant loss of IENF in the hind paws, and this decrease was significantly blocked by combined treatment with duloxetine (Figure 5 and Supplementary Figure S6). Plantar nerve fibers were denser in the control group. Long-term chemotherapy resulted in decreased nerve fiber density. Moreover, the number of nerve fibers in the duloxetine treatment group increased. These results suggested that duloxetine can improve plantar nerve injury induced by OXA and PTX.

### The Effect of Duloxetine on Expression of NF-κB, p-p38 MAPK, and p-ERK1/2 in the DRG of Mice

**FIGURE 6:**
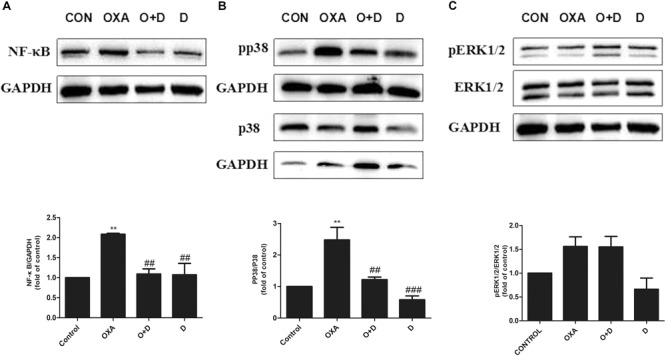
Effect of duloxetine on phosphorylation of p38 MAPK and ERK1/2 expression in the neuropathic mouse DRG following oxaliplatin treatment. **(A)** Duloxetine significantly decreased the expression of NF-κB protein in the DRG of OXA-treated mice. **(B)** Duloxetine significantly decreased the expression of pp38 protein in the DRG of OXA-treated mice. **(C)** The ratio of pERK1/2 to ERK1/2 expression was not changed significantly following oxaliplatin treatment and duloxetine did not modify p-ERK1/2 expression. (^∗∗^*p* < 0.01 vs. control, ^##^*p* < 0.01, ^###^*p* < 0.001 vs. OXA). The data are presented as the mean ± SEM (*n* = 4).

**FIGURE 7:**
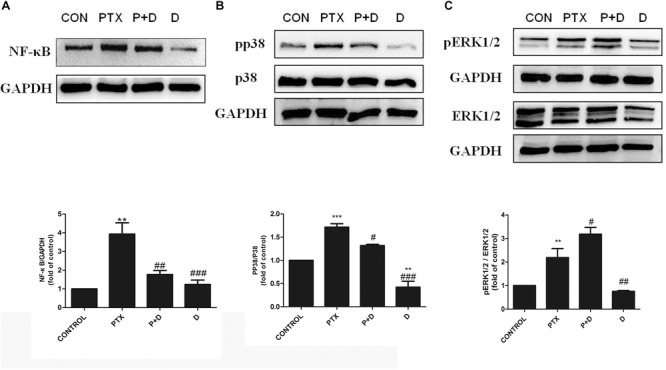
Effect of duloxetine on NF-κB, phosphorylation of p38 MAPK and ERK1/2 expression in the neuropathic mouse DRG following PTX treatment. **(A)** Duloxetine significantly decreased the expression of NF-κB protein in the DRG of PTX-treated mice. **(B)** Duloxetine significantly decreased the expression of pp38 protein in the DRG of PTX-treated mice. **(C)** The ratio of pERK1/2 to ERK1/2 expression was significantly increased after PTX treatment, but duloxetine did not modify the p-ERK1/2 expression induced by PTX. (^∗∗^*p* < 0.01 vs. control, ^∗∗∗^*p* < 0.001 vs. control, ^#^*p* < 0.05, ^##^*p* < 0.01, ^###^*p* < 0.001 vs. PTX). The data are presented as the mean ± SEM (*n* = 4).

To explore potential mechanisms of action of modulation of OXA- or PTX-induced neuropathy by duloxetine, protein expression of NF-κB, p-p38, and p-ERK1/2 was measured. As shown in Figure 6A,B and Supplementary Figure S4, protein expression of NF-κB and p-p38 was significantly increased in the OXA group compared with the control group. Co-administration with duloxetine prevented OXA-induced up-regulation of NF-κB and p-p38. However, neither OXA nor duloxetine significantly affected expression of p-ERK1/2 (Figure 6C and Supplementary Figure S4). Figure 7 and Supplementary Figure S5 shows that expression of NF-κB, p-p38 to p38 ratio, and p-ERK1/2 to ERK1/2 ratio were significantly increased in the DRG of PTX-treated mice. Thus, PTX induced the expression of NF-κB, p-p38, and p-ERK1/2 in the DRG. Moreover, these effects on NF-κB and p-p38, but not ERK1/2, were significantly reduced by duloxetine. These results suggested that NF-κB and p38 MAPK may play a role in the neuroprotective effects of duloxetine against OXA or PTX-induced peripheral neuropathy.

### Effect of Duloxetine on the Expression of Nuclear NF-κB in DRG Neuronal Cells Treated With OXA or PTX

**FIGURE 8:**
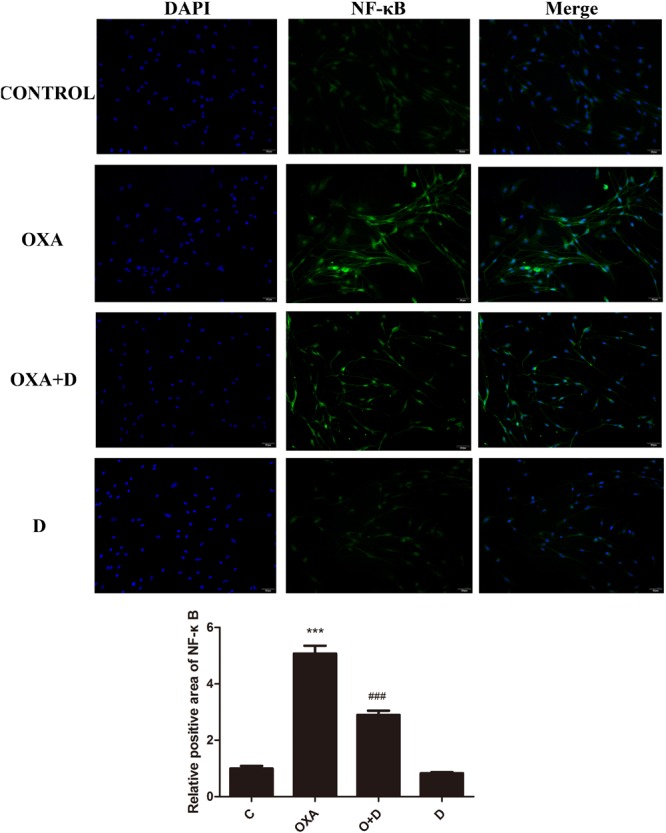
Effect of oxaliplatin and duloxetine on the expression of nuclear NF-κB in DRG neuronal cells. DRG neuronal cells were treated with (or without) OXA (3 μM) and duloxetine (3 μM) for 48 h and were double-stained with DAPI and NF-κB. The fluorescence intensity was observed using fluorescence microscopy (×200). (^∗∗∗^*p* < 0.001 vs. control; ^###^*p* < 0.001 vs. OXA).

**FIGURE 9:**
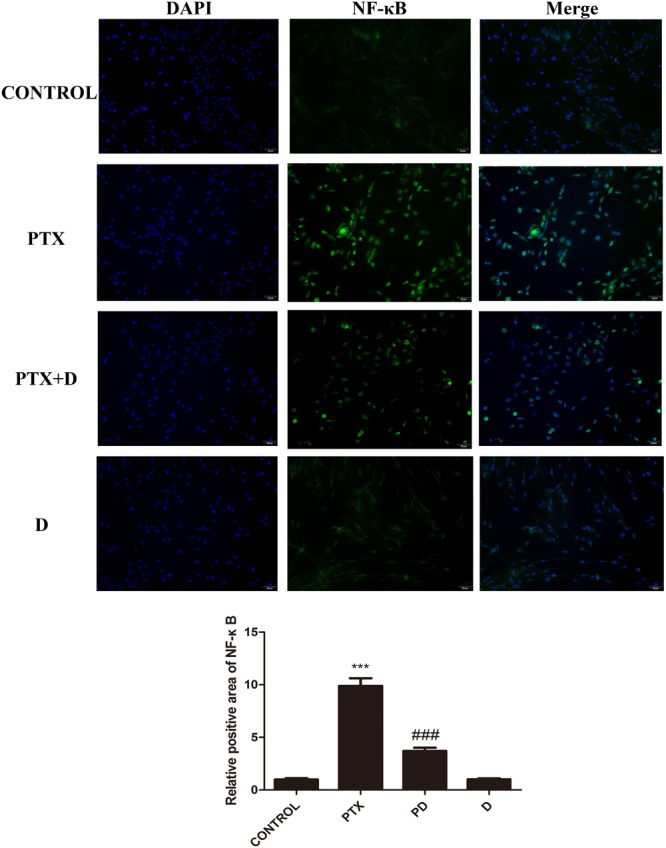
Effect of paclitaxel and duloxetine on the expression of nuclear NF-κB in DRG neuronal cells. DRG neuronal cells were treated with (or without) PTX (300 nM) and duloxetine (300 nM) for 24 h and were double-stained with DAPI and NF-κB. The fluorescence intensity was observed using fluorescence microscopy (×200). (^∗∗∗^*p* < 0.001 vs. control; ^###^*p* < 0.001 vs. PTX).

We performed immunocytochemistry to determine the expression of NF-κB using fluorescence microscopy. As seen in Figure 8, 9, treatment with OXA or PTX alone increased nuclear NF-κB levels. This increase was attenuated by co-administration with duloxetine. However, duloxetine treatment alone did not increase nuclear NF-κB levels compared to the control group.

### Duloxetine Modulated Changes in Pro-inflammatory Cytokines Induced by OXA or PTX in Neuropathic Mouse DRG Tissues

**FIGURE 10:**
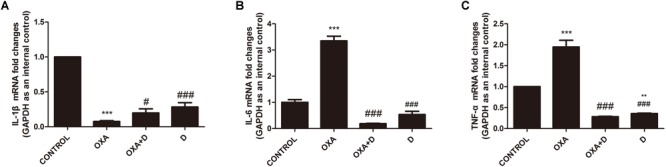
Transcript levels of the proinflammatory cytokines IL-1β, IL-6 and TNF-α in the mouse DRG. Total mRNA was extracted from mouse DRG tissues. qPCR was performed and a comparison is shown between different groups after saline or 28 days of drug treatment. **(A)** IL-1β expression was significantly decreased after OXA treatment, but duloxetine did not affect this decrease. **(B)** Duloxetine significantly decreased the expression of IL-6 mRNA in the DRG of OXA-treated mice. **(C)** Duloxetine significantly decreased the expression of TNF-α mRNA in the DRG of OXA-treated mice. (^∗∗^*p* < 0.01, ^∗∗∗^*p* < 0.001 vs. control; ^#^*p* < 0.05 vs. OXA, ^###^*p* < 0.001 vs. OXA). The data are presented as the means ± SEM (*n* = 5).

**FIGURE 11:**
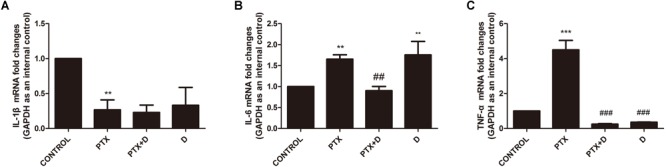
Transcript levels of the proinflammatory cytokines IL-1β, IL-6 and TNF-α in the mouse DRG. Total mRNA was extracted from mouse DRG tissues. qPCR was performed and a comparison is shown between different groups after saline or 28 days of drug treatment. **(A)** IL-1β expression was significantly decreased after PTX treatment, but duloxetine did not alter this decrease. **(B)** Duloxetine significantly decreased the expression of IL-6 mRNA in the DRG of PTX-treated mice. **(C)** Duloxetine significantly decreased the expression of TNF-α mRNA in the DRG of PTX-treated mice. (^∗∗^*p* < 0.01, ^∗∗∗^*p* < 0.001 vs. control; ^##^*p* < 0.01, ^###^*p* < 0.001 vs. PTX). The data are presented as the mean ± SEM (*n* = 5).

Chemotherapy can result in changes in expression of inflammatory factors (Makker et al., 2017). ELISA analyses demonstrated that treatment with OXA and PTX resulted in significantly increased levels of the pain-promoting inflammatory mediators IL-1β, IL-6, and TNF-α compared to the control treatment. Co-administration with duloxetine significantly attenuated OXA- and PTX-induced increases in IL-1β and NGF, but not IL-6 or TNF-α (Supplementary Table S1). Cytokine levels did not differ in response to duloxetine compared those in control mice. To further characterize inflammatory factors in neuropathic DRG tissues, we determined the mRNA expression levels of the pro-inflammatory cytokines IL-1β, IL-6, and TNF-α using real-time PCR. IL-1β expression in the DRG was significantly decreased in response to OXA or PTX treatment, and duloxetine did not alter this decrease (Figure 10, 11). However, duloxetine significantly decreased OXA- and PTX-induced increases in the mRNA expression of IL-6 and TNF-α in the DRG (Figure 10, 11). The results show that duloxetine significantly regulated the mRNA expression of IL-6 and TNF-α in the DRG, indicating that duloxetine can selectively protect the DRG.

### Duloxetine Regulated Changes in NGF Induced by OXA or PTX in Neuropathic Mice

Nerve growth factor is involved in the survival and maintenance of sensory neurons and is also a key regulator of sensitivity and sprouting of nociceptors. Our results showed that OXA and PTX significantly reduced NGF levels compared to the control group. Co-administration with duloxetine increased OXA- and PTX-induced decreases in NGF levels (Supplementary Table S1).

## Discussion

Chemotherapy-induced peripheral neuropathy causes disability and some permanent symptoms, such as pain and hypersensitivity, in up to 40% of cancer survivors (Park et al., 2013). Despite advances in chemotherapy agents, there are no FDA-approved analgesics for treatment of CIPN (Smith et al., 2015). Consequently, many cancer patients have had to terminate chemotherapy treatments and use less effective treatments. OXA and PTX are both first-line chemotherapy agents, but they can cause serious adverse reactions and sensory nerve dysfunction.

A previous study reported that duloxetine is the only drug recommended by the American Society of Clinical Oncology for treatment of chronic CIPN pain (Smith et al., 2013). In clinical trials, duloxetine was reported to have curative effects on OXA- and PTX-induced CIPN (Hirayama et al., 2015). Duloxetine is a selective inhibitor of serotonin and norepinephrine reuptake. This reuptake is believed to be involved in regulation of neuropathic pain (Piccolo and Kolesar, 2013; Okuma et al., 2016). Our results reflected that there was no effect of duloxetine on serotonin or NE level in the DRG cell culture (Supplementary Figure S7).

In this study, we used primary rat DRG neurons (from new-borns within 1 day of birth instead of embryonic day 15 rats). Based on previous reports and our experimental results (Zhu et al., 2013), we chose 3 μM and 300 nM as toxic doses for OXA and PTX, respectively. Our results showed that OXA and PTX reduced neuron mitochondrial activity and ATP levels. (Supplementary Figures S1, S2, S8) These results were consistent with those from previous studies (Xiao et al., 2011) and indicated that chemotherapy-induced mitochondrial swelling resulted in decreased cellular respiration and ATP production. However, duloxetine partially blocked these effects, resulting in increased cell survival. We found that although OXA and PTX caused significant axonal degeneration, co-treatment with duloxetine treatment enhanced neurite outgrowth. To assess whether duloxetine altered the ability of OXA and PTX to kill tumor cells, we measured the viability of HT-29 and SUM-159 cancer cell lines. Clinically, OXA or PTX is commonly used to treat colon and breast cancer patients (Cetinkaya-Fisgin et al., 2016; Areti et al., 2017). Results of this experiment showed that duloxetine did not block the chemotherapeutic effects of PTX and OXA.

In an OXA-induced chronic neuropathic pain mouse model, mechanical and cold hypersensitivities were observed. In the same mouse model PTX was also found to cause mechanical and heat hypersensitivity. Our results showed that intraperitoneal injection of duloxetine could alleviate these pain behaviors. A previous study showed that NGF participates in mechanical hyperalgesia development (Lewin et al., 2014). NGF not only plays an important role in neuronal growth and survival, but also acts as a crucial inflammatory mediator. Furthermore, NGF may have also prevented degeneration of peripheral nerves in clinical trials and experimental models of diabetic neuropathy (Apfel et al., 1994; Connor and Dragunow, 1998; Apfel, 2002). In our study, we observed that expression of NGF was decreased in CIPN model mice and found that duloxetine reversed this change and normalized the activity of neurons. Recent studies have indicated that MAPK and NF-κB signaling contributed to PTX-induced peripheral neuropathy and increased expression of pro-inflammatory cytokines, such as IL-1β and TNF-α, within the DRG (Doyle et al., 2012; Janes et al., 2014; Li et al., 2015; Zhang et al., 2016). Some studies demonstrated that the protein levels of IL-1β, and TNF-α were upregulated in rats with nerve injury. Further studies indicated mechanical allodynia can be alleviated by blocking the two proteins mentioned above (Wang et al., 2008; Li et al., 2008). We found that duloxetine effectively inhibited p38 phosphorylation and inhibited activation of NF-κB. Our results also demonstrated that duloxetine inhibited up-regulation of IL-6 and TNF-α mRNA in mouse DRG. Cell transcription and protein expression occur sequentially and follow different time courses of alteration in response to stimuli. After 4 weeks of treatment with duloxetine, transcription levels of IL-1β had decreased, and protein levels in serum were still elevated. Moreover, because the DRG is not subject to the blood-brain barrier, duloxetine can directly enter the DRG, resulting in down-regulation of IL-1β mRNA levels. Since chemotherapeutics may also affect other organs besides the DRG, duloxetine treatment may not have regulated IL-1β expression in the mice as a whole. These results suggested that duloxetine can significantly prevent OXA- or PTX- induced CIPN in a mouse model via suppression of pro-inflammatory cytokines. Previous studies have suggested that chemotherapy treatment is associated with decreased nerve fiber density (Boyette-Davis et al., 2011; Bennett et al., 2014). This may be due to reduced mitochondrial transport in peripheral sensory axons (Xiao et al., 2011). Moreover, others have suggested that decreased nerve fiber density may cause thermal hypersensitivity, mechanical hyperalgesia, and promote spontaneous discharge (Krukowski et al., 2015). Our study showed that duloxetine treatment protected against loss of IENF in the hind paws and prevented mechanical and heat hypersensitivity. Furthermore, our study showed that duloxetine administration can prevent development of peripheral neuropathy in animal models.

Our results illustrated that NF-κB and p38 MAPK in the DRG were involved in the mechanisms underlying the neuroprotective effects of duloxetine against OXA- and PTX-induced neuropathic pain. Further studies may help to elucidate the mechanisms by which duloxetine attenuates peripheral neuropathy at the genetic level.

## Conclusion

Our results suggested that duloxetine may be an effective treatment for OXA- and PTX-induced peripheral neuropathy. Furthermore, we showed that duloxetine did not reduce the chemotherapeutic efficacy of PTX or OXA. To our knowledge, this is the first report demonstrating the neuroprotective properties of duloxetine both *in vitro* and *in vivo*. In addition, we showed that duloxetine may act by blocking activation of the MAPK signaling pathways, thus preventing NF-κB activation and translocation into the nucleus. NGF may have facilitated duloxetine-induced inhibition of nerve degeneration. Our findings suggested that duloxetine may be a first-line option for treatment of CIPN.

## Ethics Statement

All experiments were performed in accordance with protocols approved by the Animal Care and Use Committee at the Nanjing University of Chinese Medicine (Approval Number AUC171001).

## Author Contributions

JZ designed and planned the experiments. LX, ZX, QZ, CY, and JM carried out the experiments. JZ, LX, and ZX wrote the manuscript. QZ, CY, and JM critical revision of the manuscript.

## Conflict of Interest Statement

The authors declare that the research was conducted in the absence of any commercial or financial relationships that could be construed as a potential conflict of interest.
