# Supplementary material for: Duloxetine, a Balanced Serotonin-Norepinephrine Reuptake Inhibitor, Improves Painful Chemotherapy-Induced Peripheral Neuropathy by Inhibiting Activation of p38 MAPK and NF-κB
Source: Front Pharmacol. 2019 Apr 9;10:365. doi: 10.3389/fphar.2019.00365 (PMC6465602; doi:10.3389/fphar.2019.00365)

**Supplemental Materials**

**Figure S1.** Prevention of oxaliplatin-induced neurotoxicity by duloxetine in vitro------------------------------------------------------------------------------------------------2

**Figure S2.** Prevention of paclitaxel-induced neurotoxicity by duloxetine in vitro------------------------------------------------------------------------------------------------2

**Figure S3.** Impact of duloxetine on body weight of ICR mice treated with or without OXA or PTX--------------------------------------------------------------------------------------3

**Figure S4.** Effect of duloxetine on phosphorylation of p38 MAPK and ERK1/2 expression in the neuropathic mouse DRG following oxaliplatin treatment-------------3

**Figure S5.** Effect of duloxetine on NF-κB, phosphorylation of p38 MAPK and ERK1/2 expression in the neuropathic mouse DRG following PTX treatment---------4

**Figure S6.** Effect of duloxetine on IENF retraction induced by OXA or PTX. --------4

**Figure S7.** Effect of duloxetine on serotonin or NE level in DRG cell culture. --------5

**Figure S8.** Effect of duloxetine on DRG-----------------------------------------------------5

**Table S1.** Cytokine changes in mice following chemotherapy and duloxetine treatments -----------------------------------------------------------------------------------------5


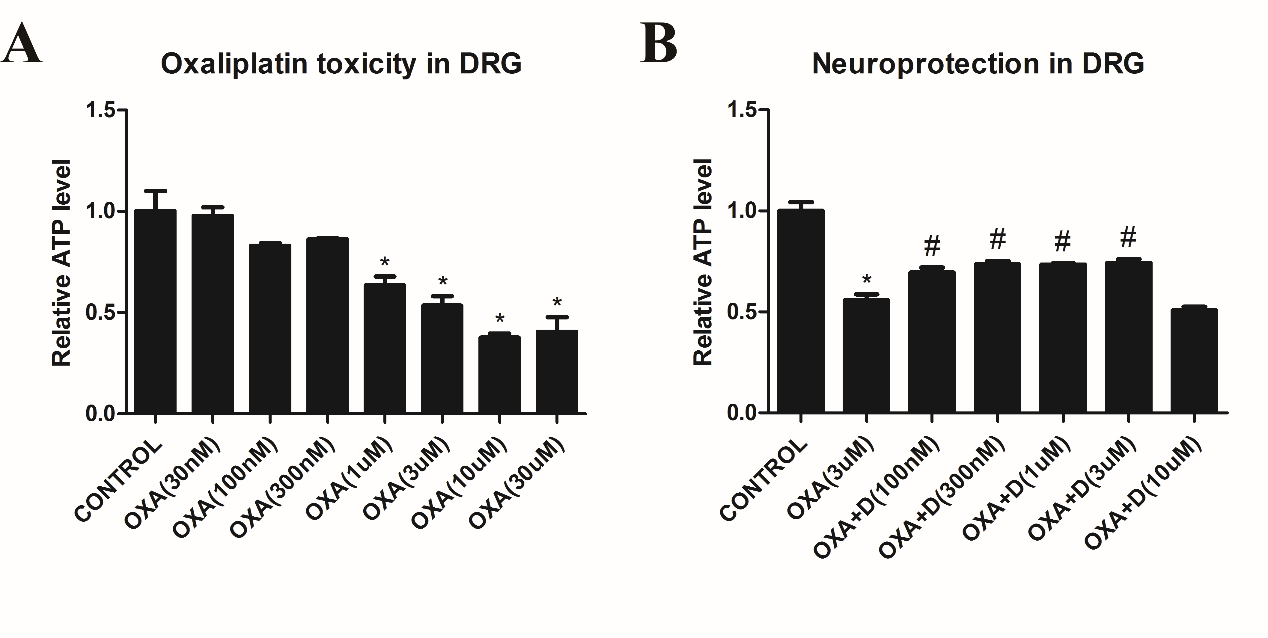


Figure S1. **Prevention of oxaliplatin-induced neurotoxicity by duloxetine in vitro. Relatived to Figure 1.**

(A) Primary rat DRG neuron cells were exposed to various concentrations of oxaliplatin for 48 hours and ATP levels was measured (*p < 0.05 vs control). (B) Primary rat DRG neuron cells were exposed to oxaliplatin with various concentrations of duloxetine for 48 hours and ATP levels were measured (* p < 0.05 vs control; # p < 0.05 vs oxaliplatin alone). All the data are presented as mean ± SEM for each experiment (n = 4).


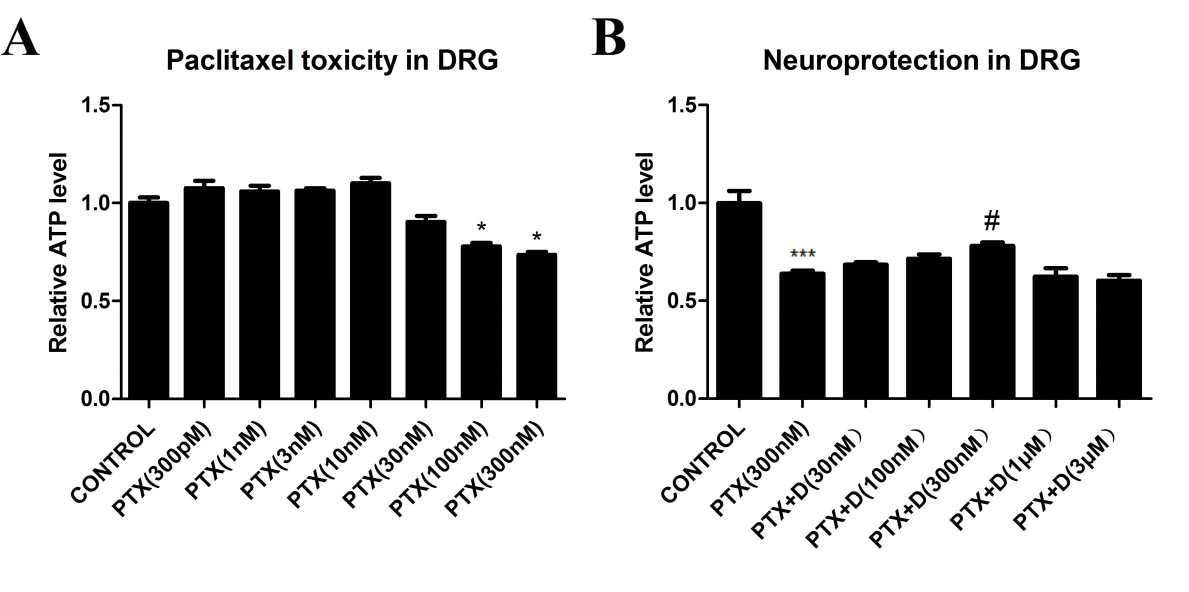


Figure S2. **Prevention of paclitaxel-induced neurotoxicity by duloxetine in vitro.** **Relatived to Figure 1.**

(A) Primary rat DRG neuron cells were exposed to various concentrations of paclitaxel for 24 hours and cell viability was measured (* p < 0.05 compared to control). (B) Primary rat DRG neuron cells were exposed to paclitaxel with various concentrations of duloxetine for 24 hours and ATP levels were measured (*** p < 0.0001 vs control; # p < 0.05 vs paclitaxel alone). All the data are presented as mean ± SEM for each experiment (n= 4).


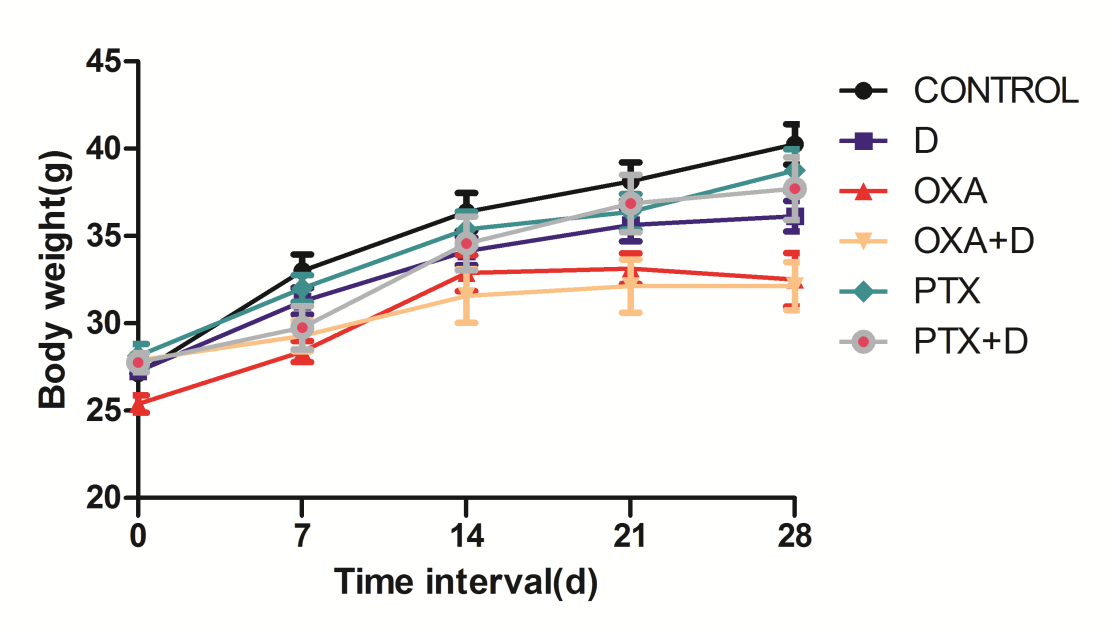


Figure S3. **Impact of duloxetine on body weight of ICR mice treated with or without OXA or PTX**.

There was no significant difference in the body weight after daily administration of duloxetine (30 mg/kg) in animals treated with vehicle or chemotherapeutic drugs (OXA and PTX).


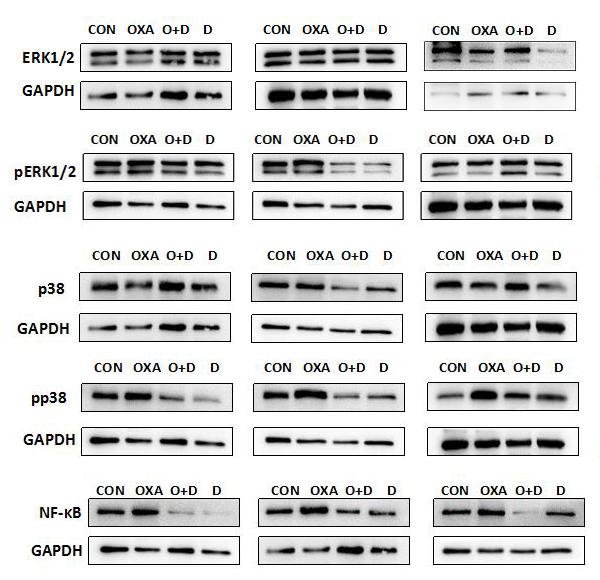


Figure S4. **Effect of duloxetine on phosphorylation of p38 MAPK and ERK1/2 expression in the neuropathic mouse DRG following oxaliplatin treatment**. **Relatived to Figure 6.**


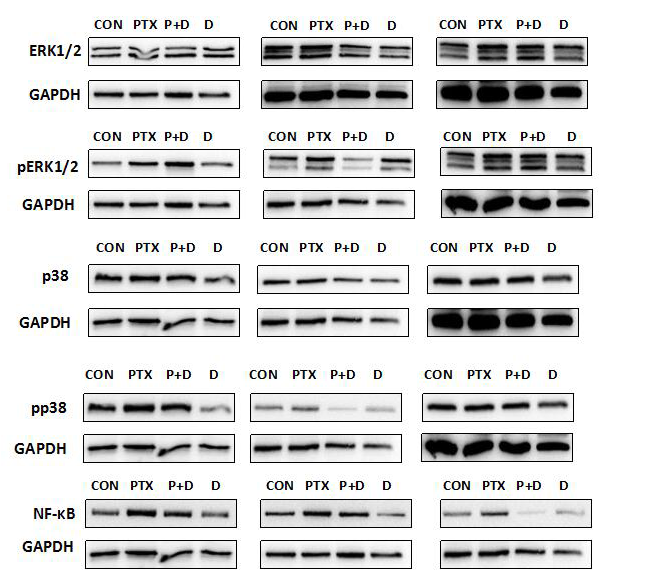


Figure S5. **Effect of duloxetine on NF-κB, phosphorylation of p38 MAPK and ERK1/2 expression in the neuropathic mouse DRG following PTX treatment**. **Relatived to Figure 7.**


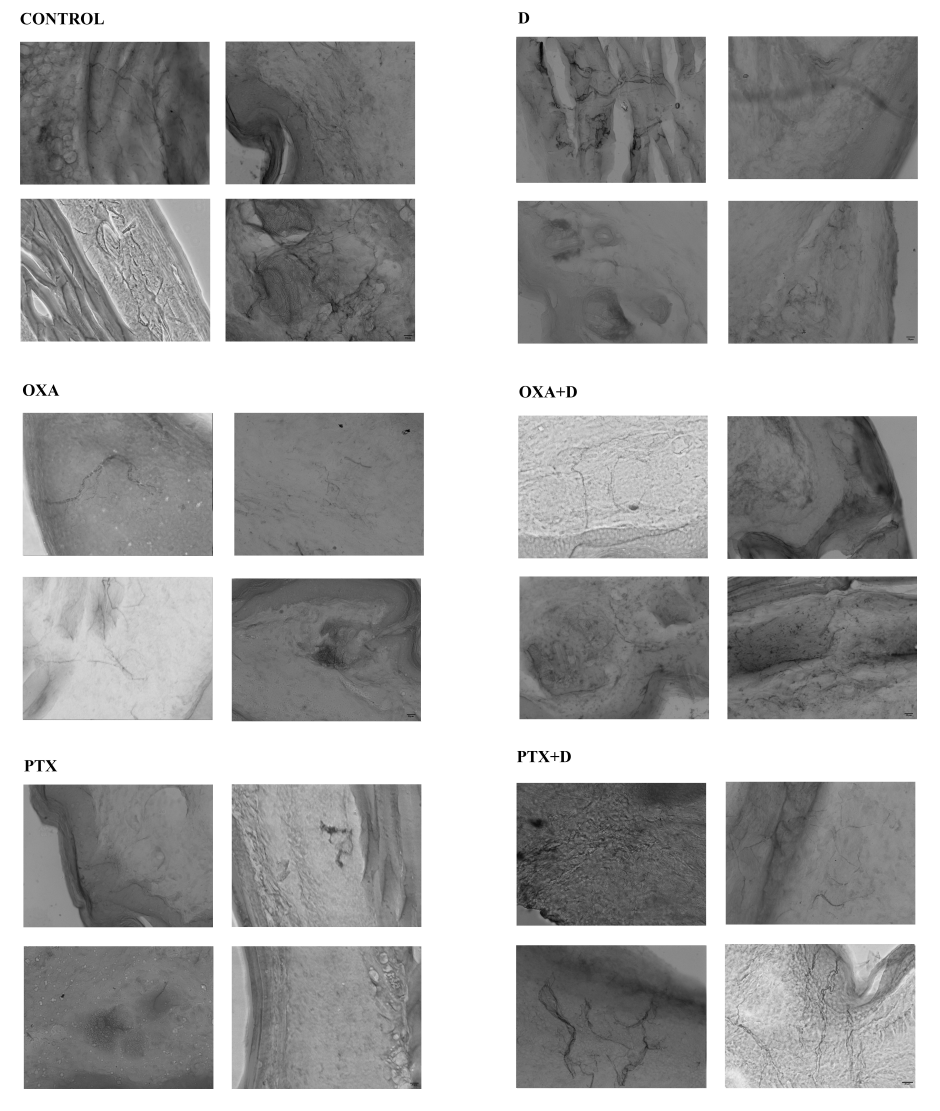


Figure S6.**Effect of duloxetine on IENF retraction induced by OXA or PTX. Relatived to Figure 5.**


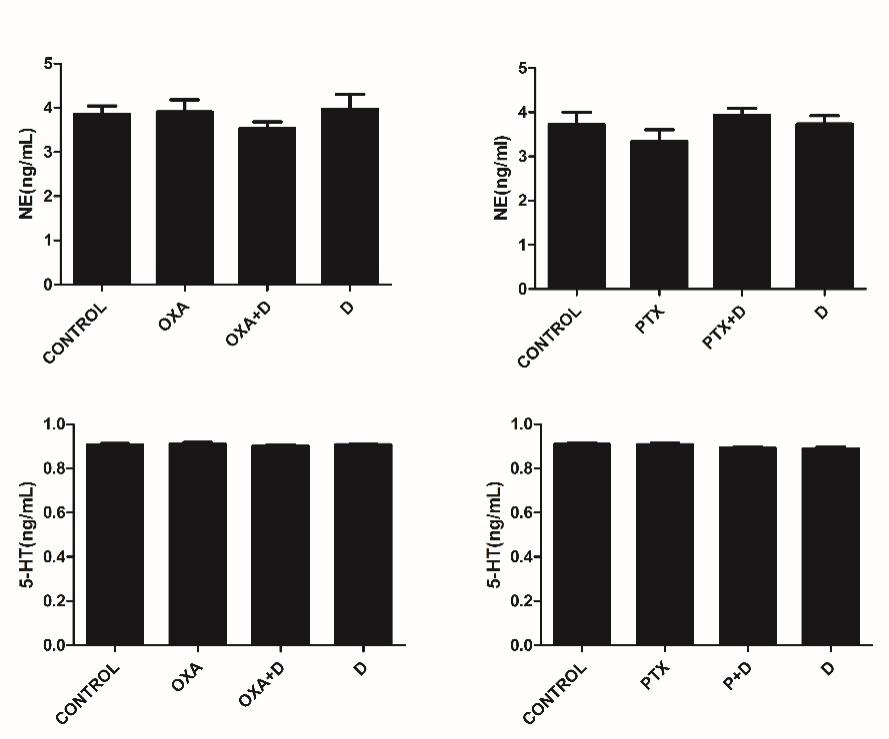


Figure S7. **Effect of duloxetine on serotonin or NE level in the DRG cell culture**. (ELISA) DRG neuronal cells were treated with (or without) PTX (300 nM) and duloxetine (300 nM) for 24 h, then serotonin and NE level were measured using Elisa kit.


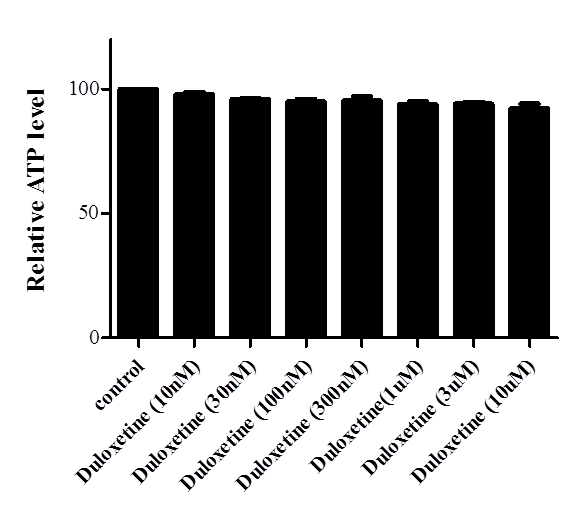


Figure S8. **Effect of duloxetine on DRG** Primary rat DRG neuron cells were exposed to various concentrations of duloxetine for 24 hours and ATP level was measured.

Table S1. **Cytokine changes in mice following chemotherapy and duloxetine treatments (pg/mL).**

The table shows the mean and SEM (standard error of the mean) for IL-1β, IL-6, TNF-α and NGF concentrations (pg/mL) in mouse blood samples. (n = 5, ** p* <0.05 vs control, *# p* <0.05 vs OXA alone, *^ p* <0.05 vs PTX alone)


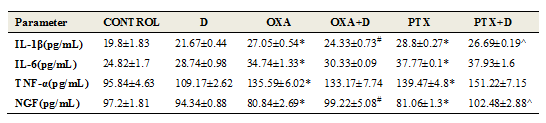

Supplement: Supplementary file 1 [file Table_1.docx]
